# Parental Substance Abuse As an Early Traumatic Event. Preliminary Findings on Neuropsychological and Personality Functioning in Young Drug Addicts Exposed to Drugs Early

**DOI:** 10.3389/fpsyg.2016.00887

**Published:** 2016-06-16

**Authors:** Micol Parolin, Alessandra Simonelli, Daniela Mapelli, Marianna Sacco, Patrizia Cristofalo

**Affiliations:** ^1^Department of Developmental Psychology and Socialization, University of PadovaPadua, Italy; ^2^Department of General Psychology, University of PadovaPadua, Italy; ^3^Therapeutic Community “Villa Renata”Venice, Italy

**Keywords:** trauma, exposure to drugs, drug-addicted youth, executive function, personality disorder

## Abstract

Parental substance use is a major risk factor for child development, heightening the risk of drug problems in adolescence and young adulthood, and exposing offspring to several types of traumatic events. First, prenatal drug exposure can be considered a form of trauma itself, with subtle but long-lasting sequelae at the neuro-behavioral level. Second, parents' addiction often entails a childrearing environment characterized by poor parenting skills, disadvantaged contexts and adverse childhood experiences (ACEs), leading to dysfunctional outcomes. Young adults born from/raised by parents with drug problems and diagnosed with a Substance Used Disorder (SUD) themselves might display a particularly severe condition in terms of cognitive deficits and impaired personality function. This preliminary study aims to investigate the role of early exposure to drugs as a traumatic event, capable of affecting the psychological status of young drug addicts. In particular, it intends to examine the neuropsychological functioning and personality profile of young adults with severe SUDs who were exposed to drugs early in their family context. The research involved three groups, each consisting of 15 young adults (aged 18–24): a group of inpatients diagnosed with SUDs and exposed to drugs early, a comparison group of non-exposed inpatients and a group of non-exposed youth without SUDs. A neuropsychological battery (Esame Neuropsicologico Breve-2), an assessment procedure for personality disorders (Shedler-Westen Assessment Procedure-200) and the Symptom CheckList-90-Revised were administered. According to present preliminary results, young drug addicts exposed to drugs during their developmental age were characterized by elevated rates of neuropsychological impairments, especially at the expense of attentive and executive functions (EF); personality disorders were also common but did not differentiate them from non-exposed youth with SUDs. Alternative multi-focused prevention and intervention programs are needed for children of drug-misusing parents, addressing EF and adopting a trauma-focused approach.

## Introduction

### The extent of the phenomenon

Substance Use Disorders are characterized by a cluster of cognitive, behavioral and physiological symptoms indicating that individuals continue using the substance despite it causes clinically and functionally significant impairment. A diagnosis of Substance Use Disorder is based on evidence of impaired control, social impairment, risky use, and pharmacological criteria (APA, 2013). Substance disorders related to alcohol and illegal drugs implicate particularly severe clinical conditions and constitute the focus of the present study.

The prevalence of SUDs related to alcohol and illegal drugs shows a systematic age-related pattern: commonly, the onset occurs during adolescence and rates peak in emerging adulthood (18–25 years; SAMHSA, 2009; Johnston et al., 2010). Adolescence is a period of specific vulnerability due to neuro-developmental immaturity (Chambers and Potenza, 2003) and to the ongoing development of inhibitory capacity (Best et al., 2009).

More than 8 million young people in the U.S. and 4.5 million in Europe live in homes with at least one parent who misuses alcohol and/or illegal drugs (Jernigan, 2001). Despite the heterogeneity of developmental outcomes (Van Voorst and Quirk, 2003; Warner et al., 2011), these children can be considered at risk for developing a SUD themselves, given the well-established intergenerational nature of the disorder (EMCDDA, 2012). Both maternal and paternal SUDs are associated with offspring's hazardous substance use and with a two-fold risk for developing SUDs; estimated prevalence rates are 53% for alcohol and 21% for illicit drugs (Chassin et al., 1999; Alati et al., 2008; Hussong et al., 2012). Controlled studies report an earlier initiation and an accelerated transition to substance disorders for exposed offspring (Chassin et al., 1996, 1999; Hussong et al., 1998, 2008; Obot et al., 2001) and these problems have long-lasting trajectories until emerging adulthood (Baer et al., 2003; Chen and Weitzman, 2005; Melchior et al., 2011). Intriguingly from a neuro-teratological perspective, prenatal substance exposure is a direct predictor of SUDs in adolescence and young adulthood, with rates of 29 and 46% respectively (Baer et al., 2003; Alati et al., 2006; Glantz and Chambers, 2006; O'Brien and Hill, 2014), but environmental characteristics contribute to the prediction of early drug use too (Warner et al., 2011). In sum, both prenatal exposure and postnatal caregivers' drug use are unique risk factors for SUDs (Delaney-Black et al., 2011).

### Pathways of parent–child transmission of SUDs

A multitude of mechanisms has been recognized accounting for SUDs parent-child transmission; they include environmental, biological and genetic factors. Meta-analytic works have demonstrated the etiologic role of genes (Malone et al., 2004; Young et al., 2004; Goldman et al., 2005; Grant et al., 2013) and substance disorders in young people have been conceived as manifestation of a general and inherited liability to disinhibitory psychopathology (McGue and Iacono, 2008; Meyers and Dick, 2010).

From a biological point of view, the intrauterine exposure to maternal drug use has subtle but enduring negative effects on the central nervous system (CNS). It affects brain architecture, in terms of under-development of prefrontal structure (Bhide, 2009; Thompson et al., 2009) and functional abnormalities (Rao et al., 2007; Sheinkopf et al., 2009), ultimately causing cognitive (Warner et al., 2006; Konijnenberg and Melinder, 2011), affective, and behavioral impairments (Jutras-Aswad et al., 2009). Although the literature on the topic is scarce and results are mixed (Engeland et al., 2013), fathers' drug use prior to conception may also influence fetal developmental and birth outcomes (Cone et al., 1995; Rubes et al., 1998; Klonoff-Cohen and Lam-Kruglick, 2001; Killinger et al., 2012; Crean and Bonduriansky, 2014).

Environmental and postnatal factors contribute to the intergenerational transmission of SUDs in an independent and equal manner (Goldman et al., 2005; O'Connor and Paley, 2009; May et al., 2013), sometimes triggering and exacerbating a preexisting genetic/biological vulnerability (Schmid et al., 2009; Vaske et al., 2009). With regard to the early childrearing environment, drug-abusing mothers tend to adopt little, inadequate, and delayed prenatal care (Singer et al., 2008; Goler et al., 2012) and they show limited knowledge of their child's developmental needs as well as wrong knowledge of the pre- and postnatal effects of drug exposure (Velez et al., 2004; Strathearn and Mayes, 2010). Their parenting style is often inadequate (Baker and Carson, 1999; Hans, 2002; Eiden et al., 2011). A similar negative style of caretaking has been recognized in drug-abusing fathers, during early child development and until the teen age (Eiden et al., 1999, 2004; Eiden and Leonard, 2000; El-Sheikh and Flanagan, 2001; El-Sheikh and Buckhalt, 2003; Edwards et al., 2004; Fals-Stewart et al., 2004; Crean and Bonduriansky, 2014). Since parents often deal with unemployment, social isolation and mental illness, they struggle to attend to their child's health, to guarantee financial support and basic needs (EMCDDA, 2012), to provide monitoring (Meyers and Dick, 2010), and emotional availability (Barnard and McKeganey, 2004). Children are likely to face multiple early traumatic and adverse experiences, such as household dysfunction and violence, parental incarceration, sexual, physical, and psychological abuse or neglect (Hussong et al., 2012; Anda et al., 2014; Taplin et al., 2014). A strong linear association exists between the number of adverse childhood experiences (ACEs), which have a cumulative effect, and a 2- to 4-fold increase in the likelihood of developing early substance problems (Tonmyr et al., 2010; Taplin et al., 2014). From a broader perspective, drug-abusing families usually live in disadvantaged neighborhoods (Daniel et al., 2009), whose characteristics potentially predispose to drug use (Kliewer and Murrelle, 2007; Meyers and Dick, 2010; Stone et al., 2012).

### The traumatic nature of being the children of substance-abusing parents

In broad terms, both prenatal and postnatal exposure to parental alcohol and illegal drug use can be conceived as forms of early maltreatment and trauma, capable of affecting children's development.

Prenatal exposure can be qualified as a biological condition of a potentially traumatic nature, since it is an event capable of causing death, injury or threatening the physical health, and welfare of the unborn child. Intrauterine drug exposure is a condition to which the child is subject passively and helplessly, perpetrated by the one who should be the primary caregiver and responsible for childcare; instead, drug-abusing women not only avoid adopting adequate and basic care, but they also actively persevere in harmful behaviors. Prenatal alcohol and illegal drug use is viewed as a behavior of “*extreme indifference*,” “*a conscious act of disregarding a risk …or a failure to exercise ordinary or due care*” (Cruz, 2005, p.8). Currently, several North-American States consider substance abuse during pregnancy to be child abuse or neglect, making it grounds for civil commitment and some of them have specifically criminalized it (Guttmacher Institute, 2015).

With regard to postnatal exposure, a childrearing environment characterized by the constant but unpredictable threat of adverse events can cause an overwhelming and chronic stress. Thus, living with drug-abusing parents may fall into the category of Complex Trauma, that is the experience of multiple, chronic and prolonged, developmentally adverse traumatic events, most often of an interpersonal nature and occurring in early development; complex trauma can lead to a wide range of symptoms (van der Kolk et al., 2005; Kearney et al., 2010). The applicability of the construct of complex trauma to people with SUDs has been demonstrated by a study reporting that 45% of adult addicts meet its criteria (Ford and Smith, 2008); moreover, each domain of complex trauma mediates the association between early adverse experiences and drug problems in young people (aged 16–24) (Rosenkranz et al., 2014).

Consistent with these conceptualizations of prenatal and postnatal trauma, findings in developmental traumatology indicate that they can both alter the neurophysiological growth of the nervous and endocrine systems, especially when they co-occur (Riley and McGee, 2005; Putnam, 2006; Henry et al., 2007; Lester et al., 2010; Enoch, 2011). Notably, stress response adaptation can be caused by relational components too, such as inadequate parental care and verbal abuse, even in the first years of life (Fisher and Gunnar, 2010; Enoch, 2011). These changes create a common physiological substrate that can disrupt child self-regulation and executive functions (EF; Woon and Hedges, 2008). As a result, these children are at high risk for developing psychopathology and, in turn, SUDs (Bailey and McCloskey, 2005; Putnam, 2006; Enoch, 2011).

Intrauterine drug exposure (Chapman et al., 2007) and the inadequate childrearing context related to parental drug-addiction (Lewis E. E. et al., 2007; Pears et al., 2008) mutually interact in affecting the development of regulatory systems (arousal, attentive and inhibitory control) and contribute to child neurobehavioral dysregulation during early development and adolescence (Dennis et al., 2006; Bada et al., 2007; Rose-Jacobs et al., 2009; Carmody et al., 2011; Fisher et al., 2011). Neurobehavioral disinhibition/dysregulation is an empirically-based construct which refers to a broad liability dimension of risk; it encompasses a wide range of disorders that commonly co-occur (Kliewer and Murrelle, 2007; Krueger et al., 2007; Hicks et al., 2012). These disorders manifest early in childhood (Cooper et al., 2003) and follow adverse developmental trajectories, potentially leading to substance misuse in adolescence and emerging adulthood (Tarter et al., 2004; Nigg et al., 2006; McNamee et al., 2008). It has been proven that effortful control is a strong mediating factor between parental drug issues and substance use in young people (Chapman et al., 2007; Lester et al., 2012).

### Executive function in children of drug-addicted parents

Given the subtle but long-lasting effects of intrauterine drug exposure on the CNS (Mayes, 2002; Li et al., 2009), neuropsychological impairments are likely to occur in offspring of drug misusing parents. Gestational exposure to cocaine, alcohol and marijuana produces a broad array of cognitive deficits in infancy (Jacobson et al., 1996; Noland et al., 2003) and preschool age (Day et al., 1994; Griffith et al., 1994; Singer et al., 2004; Frank et al., 2005; Noland et al., 2005; Behnke et al., 2006; Lewis B. A. et al., 2007). Similarly, cognitive impairments have been reported by studies on alcohol and illegal drugs during middle childhood, mainly at the expense of EF (Kodituwakku et al., 1995; Leech et al., 1999; Rasmussen et al., 2006; Goldschmidt et al., 2008; Singer et al., 2008; Eyler et al., 2009). Functional and neuroimaging evidence has detected executive function impairments in preadolescent children prenatally exposed to cocaine, alcohol and marijuana (Olson et al., 1992; Richardson et al., 2002; Savage et al., 2005). These impaired cognitive processes seem to be associated with abnormal brain structure and activity (Mayes et al., 2005; Warner et al., 2006; Rose-Jacobs et al., 2009; Day et al., 2011).

Extensive research has also been conducted in adolescence, attesting altered frontal activation, and a broad range of difficulties in higher-order activities consequent to alcohol exposure (Olson et al., 1998; Willford et al., 2001, 2004; Mattson and Roebuck, 2002). Executive function deficits are common in drug exposed adolescent offspring (Rasmussen, 2005; Rose-Jacobs et al., 2011; Grant et al., 2013), but it is worth mentioning that some studies report inconsistent results and the specific effects of prenatal exposure remain unclear (Minnes et al., 2014). In some studies prenatal cocaine-exposure was not directly related to EF in (pre)adolescence (Hurt et al., 2009; Rose-Jacobs et al., 2009), as confirmed by fMRI techniques (Hurt et al., 2008). Thus, it has been proposed that the negative effects of intrauterine cocaine and alcohol exposure on EF may be subtle (Singer et al., 2008; Richardson et al., 2011), mediated by behavioral dysregulation (Fisher et al., 2011; Lester et al., 2012) and/or they might not emerge until later age (Rose-Jacobs et al., 2009).

Beyond adolescence, neuropsychological research has not extensively explored whether adult offspring are more likely to display cognitive deficits. To the best of our knowledge, only three studies have examined young adults with prenatal exposure, specifically to alcohol and marijuana; they attest deficits in cognitive measures (Kerns et al., 1997) and altered neural activity during response inhibition (Fried and Smith, 2001; Smith et al., 2006); other research has focused on adult offspring, documenting EF impairments (Kopera-Frye et al., 1996; Kerns et al., 1997; Connor et al., 2000).

The understanding of neurocognitive outcomes also requires the consideration of postnatal and childrearing environment characteristics; it has been well-demonstrated that being raised in economically disadvantaged backgrounds and low-income households is associated with poorer EF (Mezzacappa, 2004; Lipina et al., 2005; Noble et al., 2005, 2007; Farah et al., 2006; Moilanen et al., 2010). Poor parental nurturance and insensitive parenting are capable of influencing EF in childhood (Blair and Ursache, 2011), preadolescence (Farah et al., 2008), and adolescence (Evans and Schamberg, 2009), through stress mechanisms (Mayes, 2002; Bennett et al., 2008; Singer et al., 2008).

Finally, also youth's own drug use should be taken into account for its effect on neurocognitive functioning (Tarter et al., 1995; Tapert et al., 2002; Hanson et al., 2010; Solowij and Pesa, 2010). Studies on opioids dependence have reported deficits in neurocognition (Ersche et al., 2006; Fishbein et al., 2007; Verdejo-García et al., 2007; Brand et al., 2008). However, they address adults almost exclusively, and further evidence is needed specifically addressing youth. Empirical evidence attests that family drug history and adolescent's own drug use are different risk factors for cognitive functioning (Rose-Jacobs et al., 2011) but, at the same time, they may interact with each other (Tapert and Brown, 2000). However, to our knowledge, few studies have investigated the neuropsychological functioning in young adults who are children of drug-addicted parents and who have developed a SUD themselves.

The present study aims to investigate the role of early exposure to drugs as a traumatic event, capable of affecting the psychological status of young drug addicts. In particular, it intends to provide preliminary results on neuropsychological functioning and personality profile in young adults (aged 18–24) who were diagnosed with a severe Substance Use Disorder (to the extent to be referred to residential treatment) and who were exposed to parental alcohol and illegal drug use during their prenatal and postnatal early development. In order to investigate this issue, a group of exposed drug addicts was compared to a group of drug addicts who were not exposed to drugs, and to a group of peers who did not report drug problems. On the basis of previous literature, it can be expected that exposed young drug addicts show greater neuropsychological impairments and personality disorders, given the exposure to the toxic effects of drugs and/or to the adverse childrearing environment consequent to parental drug misuse. In sum, parental drug use could be an additional factor that makes the clinical condition of young drug addicts even more severe than the one of the non-exposed counterpart.

## Materials and methods

The present study adopted a descriptive, cross-sectional and correlational perspective for examining the neuropsychological and personality features of a group of young adults with SUDs, offspring of parents with drug problems.

### Participants and procedure

Participants with SUDs were recruited from the Therapeutic Community “Villa Renata” in Venice, Italy. The following inclusion criteria were adopted: (a) meeting DSM-IV-TR (APA, 2000) criteria for Substance Use Disorder; (b) having spent < 3 months in the treatment facility; (c) age ranging from 18 to 24 years. 30 inpatients, aged 18–24, constituted the clinical group of drug addicts. The assessment took place on average 1.6 months after patients' admission. At the time of recruitment, the participants had been abstinent from drugs for, on average, 3.2 months. Drug-addicted inpatients were categorized according to the criteria of having or not being exposed to parental substance misuse, as reported by records provided by the outpatient mental health services that referred the patient to the Therapeutic Community; two different groups of 15 inpatients each were obtained. The “exposed” group included young people with SUDs who had at least one biological parent with alcohol and/or illegal drug problems (cocaine, opioids, marijuana), either at the time of conception or/and during early development. The exposed participants were 66.7% male and their mean age was 20.40 (±2.2). Dichotomous variables were computed indicating the type of exposure (0 = absent; 1 = present); 73.3% of the subjects were prenatally and biologically exposed, because of substance misuse by the mother during gestation (54.5%) and/or by the father at the time of conception (63.3%). With regard to postnatal exposure, 93.3% of the exposed group experienced an upbringing environment characterized by primary caregivers' substance related problems; 57.1% were exposed to maternal and 71.4% to paternal substance misuse during their development. The “non-exposed” inpatients were 53.3% male; the mean age was 21.13 years (±2.3) (see Table 1). Most of young drug addicts (both exposed and non-exposed) were characterized by low levels of education and were unemployed. The majority of subjects indicated heroin as the primary substance of abuse. However, both non-exposed and exposed inpatients were mainly poly-drug users, having used alcohol and cannabis (100%), cocaine (93.3%), and different synthetic drugs such as MDMA (80 and 66.7%, respectively), and ketamine (73.3%). In addition, the use of non-prescribed drugs was quite common, with 53.3% of non-exposed and 50% of exposed subjects using methadone procured illegally and 46.7 and 33.3% using psychiatric drugs. On average, the onset of drug-related problems occurred during adolescence at 15–16 years.

**Table 1:** **Demographics of participants**.

	**Drug addicts (clinical group)**		**Non-drug**
	**Exposed**	**Non-exposed**	
	**% (*N*) or *M* (*SD*)**	**% (*N*) or *M* (*SD*)**	**% (*N*) or *M* (*SD*)**
Gender: female	33.3 (5)	46.7 (7)	46.7 (7)
Age	20.40 (±2.2)	21.13 (±2.3)	20.3 (±2.1)
High school degree	13.3 (2)	46.7 (7)	53.3 (8)
School drop-out	80 (12)	73.7 (7)	
Years of education	9.66 (±2.29)	10.27 (±1.49)	11 (±1.2)
Unemployment	86.7 (13)	53.3 (8)	
Poly-drug use	80 (12)	80 (12)	
Primary drug of abuse: heroin	66.7 (10)	86.7(13)	
Use of synthetic drugs	73.3 (11)	80 (12)	
Age of SUDs onset	15.3 (1.8)	16 (2.3)	
Age of first contact SUDs services	18.3 (2.3)	18.5 (2.7)	
SUDs-related diseases	33.3 (5)	13.3 (2)	
Pharmacotherapy	93.3 (14)	53.3 (8)	
Replacement therapy	46.7 (7)	66.7 (10)	
Traumatic events	80 (11)	60 (9)	
Early abuse/maltreatment	40 (6)	40 (6)	
Attachment disruptions	53.3 (8)	33.3 (5)	
Parental psychiatric problems	13.3 (2)	6.7 (1)	
Prenatal exposure	73.3 (11)		
Intrauterine exposure	54.5 (6)		
Paternal SUDs at conception	63.6 (7)		
Maternal and Paternal	18.2 (2)		
prenatal exp.			
Postnatal exposure	93.3 (14)		
Maternal SUDs	57.1 (8)		
Paternal SUDs	71.4 (10)		
Maternal and paternal SUDs	28.6 (4)		
Prenatal and postnatal exposure	66.7 (10)		

*Exposed, subjects exposed to drugs early; Non-exposed, subjects not exposed to drugs early; Non-drug, subjects of the comparison group*.

Traumatic events were computed in a categorical index, which included the experience of attachment disruptions (parental loss or abandonment, primary caretaker change, and foster care, early separation from the caregiver) and/or maltreatment or abuse (sexual, physical abuse, exploitation) in childhood and adolescence. A high percentage of exposed and non-exposed participants, 60 and 80%, respectively, faced adverse events during their developmental period, often perpetuated by relatives.

The comparison group was recruited from vocational schools in an area near Venice and it included 15 young adults (*N* = 15), aged 20.3 (±2.1); 53.3% were male. The comparison subjects did not report drug use or traumatic or adverse events in their development^1^.

### Instruments

- Brief Neuropsychological Examination-2 [*Esame Neuropsicologico Breve-2*] (ENB-2; Mondini et al., 2011). This is a comprehensive neuropsychological battery ideated and standardized for the Italian population. It includes 16 subtests (Digit span, Immediate and Delayed recall prose memory, Interference memory at 10 and 30 s, a Trial making test parts A and B, Token test, Word phonemic fluency test, Abstract reasoning test, Cognitive estimation test, Test of overlapping figure, Spontaneous drawing, Copy drawing, Clock drawing, and Ideative and ideomotor praxis test). The ENB-2 allows to investigate several cognitive domains: attention, executive functioning, perception, praxis abilities, and comprehension. The battery provides both an assessment of the single cognitive tasks and a total score (Global cognitive index) indicating the overall cognitive profile. Age (15–20, 21–30 years) and education (lower than 9 years and higher) are the two criteria used to identify subgroups of individuals and their respective normative scores. The 5th percentile was used to determine cut-off scores for each subgroups; according to the cut-off score, the performance is classified in three categories: below average (impaired), at the limit, and average (normative). The battery shows good psychometric characteristics, revealing good differential validity in discriminating normative and clinical groups and sufficient test-retest reliability (range from 0.57 to 0.97) (Mondini et al., 2003, 2011).
- The Shedler–Westen Assessment Procedure (SWAP-200; Westen and Shedler, 1999a,b) is a set of 200 descriptive statements (items) regarding adult personality aspects. It is based on the Q-Sort method, with fixed score distribution, and it requires the clinician to sort the items into eight categories based on their applicability to the patient, from 7 (*highly descriptive*) to 0 (*not descriptive*). The major advantage of the present assessment is that it relies on an external observer's judgment instead of self-reporting, which is subject to a number of biases. The SWAP-200 assessment provides: (a) a personality diagnosis expressed as the matching of the patient's description with 10 prototypical descriptions of *DSM-IV* personality disorders (standardized score named PD-T); according to DSM, the 10 personality disorders are grouped into three clusters (A,B,C); (b) a personality diagnosis based on the matching of the patient with 11 Q-factors of personality derived empirically (standardized scores named Q-T); (c) a dimensional profile of healthy and adaptive functioning. The presence of one or more personality disorders is determined when the patient's PD-T and/or Q-T are ≥60 and the adaptive functioning scale is ≤ 60; if the score ranges from 55 to 60, then subclinical traits of that personality disorder or style are present. In sum, the SWAP-200 provides both categorical and dimensional diagnoses. The reliability of SWAP-200 personality descriptions range from 0.75 to 0.89 (Shedler and Westen, 1998; Westen and Muderrisoglu, 2003; Marin-Avellan et al., 2005) and scores correlate with several external criterion measures (e.g., Westen and Shedler, 1999a; Westen and Muderrisoglu, 2003; Westen and Weinberger, 2004). The study by Blagov et al. (2012), after reviewing empirical evidence on the SWAP-200, attests its validity and reports new test-retest reliability data (median coefficient > 0.85). To our knowledge, an Italian translation of the SWAP-200 is currently available (Lingiardi et al., 2006; Shedler et al., 2014). Given the need for a clinician's judgment to complete the SWAP-200, this assessment was not administered to the comparison group.
- The Symptom Checklist-90-Revised (SCL-90-R; Derogatis, 1994). It is a self-report measure assessing 90 clinical symptoms on a 5-point Likert scale, ranging from 0 (*not at all*) to 4 (*extremely*). The symptoms are factored into nine psychiatric dimensions (depression, anxiety, somatization, obsessive-compulsive behavior, interpersonal sensitivity, hostility, phobic anxiety, psychoticism, and paranoid ideation), plus altered appetite and disturbed sleep. The instrument provides three global scores: the Global Stress Index (GSI) indicating the general psychological distress of the individual; the Positive Symptom Total (PST), revealing the number of symptoms the respondent has endorsed to any degree; the Positive Symptom Distress Index (PSDI), a measure of distress intensity. The psychometric properties of the original version of the checklist show acceptable levels of internal consistency (ranging from 0.77 to 0.90), test-retest reliability (ranging from 0.68 to 0.90) and convergent and discriminant validity (Derogatis, 2011). However, despite the extensive and widespread application of the instrument, some studies have questioned its factorial invariance across different samples (Cyr et al., 1985; Prunas et al., 2012). The Italian translation and adaptation of the SCL-90-R (Sarno et al., 2011) show adequate results for Principal Component Analysis (a single factor explains 65.22% of the variance) and internal consistency with Cronbach's alpha (from 0.68 to 0.87 for the nine dimensions and = 0.97 for the GSI score).

## Results

Data were analyzed using the IBM Statistical Package for the Social Sciences (SPSS) 23.0. The qualitative analysis was carried out using descriptive statistics (frequencies, mean scores, and percentages), while for the quantitative analysis, due to the small number of subjects, non-parametric tests were applied (the Mann–Whitney U test, Pearson's Chi-Square test, and Spearman's Rho correlation).

### Descriptive analysis

Results on the psychological distress assessed by the self-report SCL-90-R displayed that more than a half of both exposed and non-exposed young inpatients showed clinically significant scores on the global symptomatological profile, with GSI scores above the clinical cut-off for 53.3 and 66.7% of individuals respectively, as shown in Table 2. Similarly, the evaluation of symptoms' number (PST) and intensity (PSDI) showed high values for a considerable number of subjects in both groups, especially the non-exposed one. The majority of drug addicts biologically or environmentally exposed to parental SUDs fall in the clinical range for anxiety and depression, while most of the non-exposed individuals revealed high levels of depression, hostility, and psychoticism.

**Table 2:** **Symptomatological profile at SCL-90-R**.

**Scales**	**Drug addicts (clinical group)**								**Non-drug**			
	**Exposed**				**Non-exposed**							
	**min**	**max**	***M* (*SD*)**	**Clinical % (*N*)**	**min**	**max**	***M* (*SD*)**	**Clinical % (*N*)**	**min**	**max**	***M* (*SD*)**	**Clinical % (*N*)**
Somatization	39	76	54.20 (12.04)	40.0 (6)	39	76	56.40 (14.62)	53.3 (8)	37	72	50.07(10.71)	26.7 (4)
Obsessionality	37	72	54.53 (11.82)	46.7 (7)	37	75	54.87 (12.58)	53.3 (8)	37	61	47.20 (8.15)	20.0 (3)
Inter. sensitivity	40	76	54.93 (13.59)	40.0 (6)	38	74	56.20 (11.95)	53.3 (8)	38	60	46.27 (8.22)	20.0 (3)
Depression	43	76	58.33 (11.88)	60.0 (9)	44	76	62.80 (11.51)	66.7 (10)	35	63	48.07 (7.96)	26.7 (4)
Anxiety	40	76	59.20 (12.68)	53.3 (8)	43	76	59.13 (13.58)	53.3 (8)	39	64	49.40 (8.71)	33.3 (5)
Hostility	41	76	58.13 (12.14)	46.7 (7)	39	76	60.67 (13.47)	66.7 (10)	38	60	47.27 (6.74)	20.0 (3)
Phobic anxiety	43	76	55.67 (13.11)	40.0 (6)	43	76	49.93 (10.10)	20.0 (3)	42	76	48.40 (9.61)	20.0 (3)
Paranoid ideat.	39	76	55.60 (15.22)	46.7 (7)	36	76	59.60 (13.37)	60.0 (9)	36	63	51.27 (8.79)	46.7 (7)
Psychoticism	42	76	57.00 (13.33)	40.0 (6)	42	76	60.93 (11.42)	66.7 (10)	40	56	47.47 (5.12)	13.3 (2)
GSI	39	76	57.87 (12.99)	53.3 (8)	41	76	60.33 (12.60)	66.7 (10)	39	62	48.60 (7.85)	26.7 (4)
PST	35	73	55.20 (11.30)	53.3 (8)	37	75	55.87 (11.91)	60.0 (9)	33	65	48.33(10.49)	33.3 (5)
PSDI	42	76	58.40 (14.01)	53.3 (8)	48	76	63.73 (9.64)	80.0 (12)	30	63	49.67 (8.33)	26.7 (4)

*Clinical, percentages of individuals who obtained scores above the normative cut-off; GSI, Global Severity Index; PST, Positive Symptom Total; PSDI, Positive Symptom Distress Index*.

The SWAP-200 procedure indicated that Personality Disorders (PDs) were frequent among young drug addicts of both groups. With respect to DSM classification, 66.7% of exposed and 46.7% of non-exposed drug addicts showed at least one PD diagnosis. The primary diagnosis referred more frequently to cluster B (for 46.7% of the exposed subjects and 26.7% of the non-exposed ones), while in both groups 13.3% of the individuals received a cluster A primary diagnosis and 6.7% a cluster C primary diagnosis. Histrionic Personality Disorder was the most common primary diagnosis, with 20% of the exposed patients and 13.3% of the non-exposed young addicts. Considering the presence of both traits and disorders, the exposed group showed high rates of Borderline Personality features, while the non-exposed group showed high rates of Dependent and Schizotypal characteristics. In relation to the Q-factor categorization, a PD diagnosis was detected in 80% of the exposed subjects and in 60% of their counterpart. The primary diagnosis were Dependent personality style for the former (33.3%) and Histrionic (13.3%) for the latter. Depressive, Dysregulated and Antisocial components were frequent in the exposed group, while non-exposed drug addicts showed high rates of Antisocial and Hostile functioning. The adaptive functioning resulted inadequate for 73.3% of individuals with parental SUDs and for 93.3% of inpatients without past drug exposure (Table 3).

**Table 3:** **Personality traits and disorder scores at the SWAP-200 for the clinical groups**.

		**Exposed**						**Non-exposed**					
	**Personality**	**min**	**max**	***M* (*SD*)**	**Trait % (*N*)**	**Disorder % (*N*)**	**Primary % (*N*)**	**min**	**max**	***M* (*SD*)**	**Trait % (*N*)**	**Disorder % (*N*)**	**Primary % (*N*)**
PD-T	PD					66.7 (10)						46.7 (7)	
	Cluster A						13.3 (2)						13.3 (2)
	Paranoid	34.35	72.00	46.95 (11.0)	6.7 (1)	13.3 (2)	6.7 (1)	38.21	60.73	48.61 (6.79)	13.3 (2)	6.7 (1)	6.7 (1)
	Schizoid	33.07	60.17	44.25 (7.64)	–	6.7 (1)	6.7 (1)	33.39	59.83	46.27 (8.15)	13.3 (2)	–	–
	Schizotypal	31.96	58.29	47.15 (7.60)	20.0 (3)	–	–	35.55	60.49	48.21 (7.67)	20.0 (3)	6.7 (1)	6.7 (1)
	Cluster B						46.7 (7)						26.7 (4)
	Antisocial	41.09	62.93	51.16 (7.21)	6.7 (2)	20.0 (3)	6.7 (1)	43.35	68.24	52.11 (5.97)	13.3 (2)	6.7 (1)	6.7 (1)
	Borderline	41.74	67.06	56.60 (7.55)	26.7 (4)	33.3 (5)	13.3 (2)	47.87	66.48	53.55 (5.17)	6.7 (1)	13.3 (2)	6.7 (1)
	Histrionic	43.27	65.57	55.63 (6.54)	33.3 (5)	26.7 (4)	20.0 (3)	39.93	66.11	53.35 (6.77)	13.3 (2)	20.0 (3)	13.3 (2)
	Narcissistic	41.02	65.49	50.45 (7.91)	13.3 (2)	13.3 (2)	6.7 (1)	40.24	62.13	51.25 (6.29)	20.0 (3)	6.7 (1)	
	Cluster C						6.7 (1)						6.7 (1)
	Avoidant	35.83	54.13	45.07 (5.50)	–	–	–	35.92	56.99	46.03 (5.67)	6.7 (1)	–	–
	Dependent	36.87	64.04	51.39 (7.04)	26.7 (4)	6.7 (1)	6.7 (1)	45.25	59.44	50.20 (4.76)	26.7 (4)	–	–
	Obsessive	32.64	49.85	41.98 (4.88)	–	–	–	35.21	60.05	46.85 (7.44)	13.3 (2)	6.7 (1)	6.7 (1)
Q-T	PD					80.0 (12)						60.0 (9)	
	Antisocial	40.85	63.67	51.36 (7.35)	13.3 (2)	20.0 (3)	6.7 (1)	42.97	70.48	52.60 (6.31)	26.7 (4)	6.7 (1)	6.7 (1)
	Schizoid	32.25	59.46	44.03 (7.82)	6.7 (1)	–	–	34.69	60.98	46.61 (8.10)	6.7 (1)	6.7 (1)	–
	Paranoid	35.15	69.04	48.25 (10.47)	13.3 (2)	13.3 (2)	13.3 (2)	38.04	64.67	48.16 (6.90)	13.3 (2)	6.7 (1)	6.7 (1)
	Obsessive	33.15	65.34	46.85 (7.90)	6.7 (1)	6.7 (1)	–	40.22	58.34	49.51 (5.65)	20.0 (3)	–	–
	Histrionic	41.66	65.76	55.02 (7.17)	13.3 (2)	33.3 (5)	–	34.72	67.57	53.92 (7.97)	20.0 (3)	20.0 (3)	13.3 (2)
	Narcissistic	32.14	57.87	46.27 (7.39)	13.3 (2)	–	–	36.63	69.63	46.82 (8.25)	–	6.7 (1)	6.7 (1)
	Avoidant	36.74	57.78	45.01 (6.51)	6.7 (1)	–	–	36.89	59.70	46.21 (7.24)	13.3 (2)	–	–
	Depressive	37.78	71.30	51.90 (8.76)	26.7 (4)	13.3 (2)	13.3 (2)	40.37	58.86	49.95 (5.13)	20.0 (3)	–	–
	Dysregulate	30.79	69.62	51.70 (9.40)	20.0 (3)	13.3 (2)	13.3 (2)	41.44	60.93	48.57 (6.43)	6.7 (1)	13.3 (2)	6.7 (1)
	Dependent	43.74	69.67	56.23 (8.06)	20.0 (3)	33.3 (5)	33.3 (5)	45.47	68.25	54.35 (5.55)	13.3 (2)	13.3 (2)	6.7 (1)
	Hostile	30.61	63.35	45.50 (9.43)	6.7 (1)	13.3 (2)	–	37.75	61.45	49.83 (7.47)	20.0 (3)	13.3 (2)	6.7 (1)
Adaptive functioning		37.50	72.85	49.50 (9.44)	13.3 (2)	73.3 (11)		40.53	56.12	49.50 (4.91)	6.7 (1)	93.3 (14)	

*Trait, scores ranging from 55 to 60, indicating subclinical traits of the personality style; Disorder, scores ≥ 60, indicating the presence of a PD of clinical significance; Primary, primary diagnosis, that is the personality disorder with highest score; PD, presence of a Personality Disorder diagnosis*.

With regard to neuropsychological functioning, the results of the ENB-2 showed that 80% of inpatients with parental drug exposure had an impaired global cognitive profile, considering both those whose Global cognitive index reached the limits of the normative performance and those who had fully impaired neuropsychological functioning. This rate seemed to exceed the one of the non-exposed drug addicts. Observing the performance of the single tests of the ENB-2 battery, deficits occurred primarily in the domain of executive function, with three subtests displaying a high percentage of impairments, namely: Tmt-B (73.3%) (a test of attention and task switching, providing information about visual search speed, mental flexibility, and EF); Cognitive estimation test (assessing the capacity of answering a question for which relevant knowledge, but not the specific answer, is available) and Clock drawing test (which taps into a wide range of cognitive abilities including EF) with rates of 46.6% each. Other impairments occurred frequently in the memory domain, specifically the Immediate Recall test and the Interference test at 30 s. The non-exposed individuals showed a similar pattern of executive and memory problems, but with lower rates of altered performance. Notably, 66.7% of the non-exposed young addicts demonstrated no or one deficit in the executive domain, while those in patients who were exposed to parental SUDs during development had an impaired performance in at least two tests (maximum five) in 73.3% of cases (see Table 4).

**Table 4:** **Percentages of patients with altered neuropsychological functioning**.

**Cognitive ability**	**Drug addicts (clinical group)**		**Non-drug**
	**Exposed**	**Non-exposed**	
	**% (*N*)**	**% (*N*)**	**% (*N*)**
**ATTENTION**			
Tmt-A	33.3 (5)	6.7 (1)	–
Tmt-B	73.3 (11)	33.3 (5)	–
**MEMORY**			
Digit span	6.7 (1)	6.7 (1)	6.7 (1)
Immediate recall	46.7 (7)	33.3 (5)	40.0 (6)
Delayed recall	33.3 (5)	20.0 (3)	33.3 (5)
Interference 10s	33.3 (5)	20.0 (3)	13.3 (2)
Interference 30s	46.7 (7)	20.0 (3)	13.3 (2)
**EXECUTIVE FUNCTIONS**			
Tmt-B	73.3 (11)	33.3 (5)	–
Cognitive estimation	46.7 (7)	33.3 (5)	–
Abstract reasoning	33.3 (5)	6.7 (1)	20.0 (3)
Phonemic fluency	26.7 (4)	13.3 (2)	33.3 (5)
Clock drawing	46.7 (7)	26.7 (4)	26.7 (4)
Overlapping figures	13.3 (2)	6.7 (1)	6.7 (1)
**PERCEPTION**			
Spontaneous drawing	–	6.7 (1)	6.7 (1)
Copy drawing	33.3 (5)	26.7 (4)	33.3 (5)
Praxis Ability			
Ideomotory praxis	6.7 (1)	–	6.7 (1)
**COMPREHENSION**			
Token test	13.3 (2)	–	–
Global Index	80.0 (12)	20.0 (3)	20.0 (3)
Number of impaired EF			
0–1	26.7 (4)	66.7 (10)	40.0 (6)
2–5	73.3 (11)	33.3 (5)	60.0 (9)

*Global Index, index of the overall cognitive profile; Number of impaired EF, number of tasks assessing executive functions showing an impaired performance*.

### Comparing the two clinical groups and the comparison group

First, concerning the important socio-demographical variables capable of influencing the performance at the neuropsychological battery (cfr. Mondini et al., 2011), the three groups were compared for age and years of education. The Kruskal–Wallis test did not report significant differences (age *X*^2^ = 1.643, *p* = 0.440, ns), (education *X*^2^ = 4.788, *p* = 0.091, ns). Also, the use of the Mann–Whitney test confirmed that the comparison group was similar in age both to the exposed (*z* = −0.085, *p* = 0.935, ns) and to the non-exposed group (*z* = −1.268, *p* = 0.217, ns). With regard to years of education, there was no significant difference according to the Bonferroni correction between the comparison group and the exposed individuals (*p* = 0.05/2 = 0.025) (*z* = 2.113, *p* = 0.041) and with the non-exposed subjects (*z* = −1.390; *p* = 0.187, ns).

**Figure 1:**
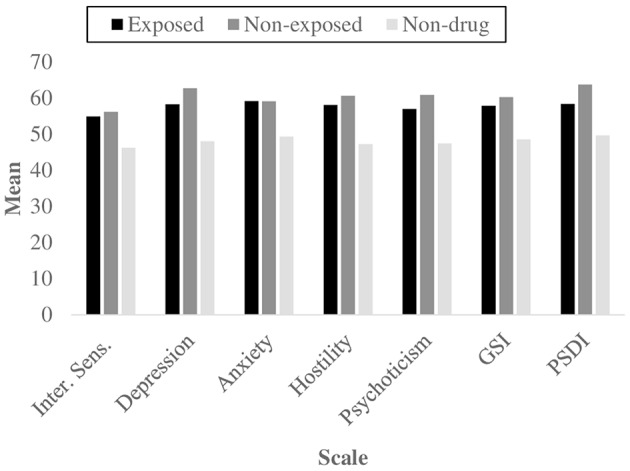
**Mean scores for the symptomatological scales and global indexes that showed significant differences using Kruskal–Wallis and Mann–Whitney's U tests, as a function of group**.

Second, the symptomatological features of the three groups were analyzed in order to ascertain if the comparison group did not display any significant clinical status, as opposed to the two groups with SUDs. The Kruskal–Wallis test by ranks revealed a significant difference among the three independent samples for the global indexes GSI (*X*^2^ = 7.247, *p* = 0.027) and PSDI (*X*^2^ = 9.334, *p* = 0.009), and for 5 psychiatric dimensions: interpersonal sensitivity (*X*^2^ = 6.416, *p* = 0.040), depression (*X*^2^ = 11.694, *p* = 0.003), anxiety (*X*^2^ = 6.006, *p* = 0.050), hostility (*X*^2^ = 19.377, *p* = 0.009) and psychoticism (*X*^2^ = 10.709, *p* = 0.005) (Figure 1); results for depression and psychoticism remained significant when the Bonferroni correction was used. While the chi-square test did not report any significant results, the Mann–Whitney U test reported that the comparison group differed from the exposed group for the Global Stress Index, GSI (*z* = −1.96, *p* = 0.049) and for the symptomatic dimensions of depression (*z* = −2.35, *p* = 0.019), anxiety (*z* = −2.12, *p* = 0.033), hostility (*z* = −2.56, *p* = 0.010) (significant also for Bonferroni correction) and psychoticism (*z* = −1.99, *p* = 0.049). It also significantly differed from the non-exposed drug addicts group, for the GSI (*z* = −2.55, *p* = 0.010), PSDI (*z* = −3.28, *p* = 0.001) and 5 scales, interpersonal sensitivity (*z* = −2.41, *p* = 0.015), depression (*z* = −3.14, *p* = 0.001), anxiety (*z* = −2.10, *p* = 0.037), hostility (*z* = −2.64, *p* = 0.008) and psychoticism (*z* = −3.31, *p* = 0.001). The use of Bonferroni test revealed significant results for PSDI, depression and psychoticism.

**Figure 2:**
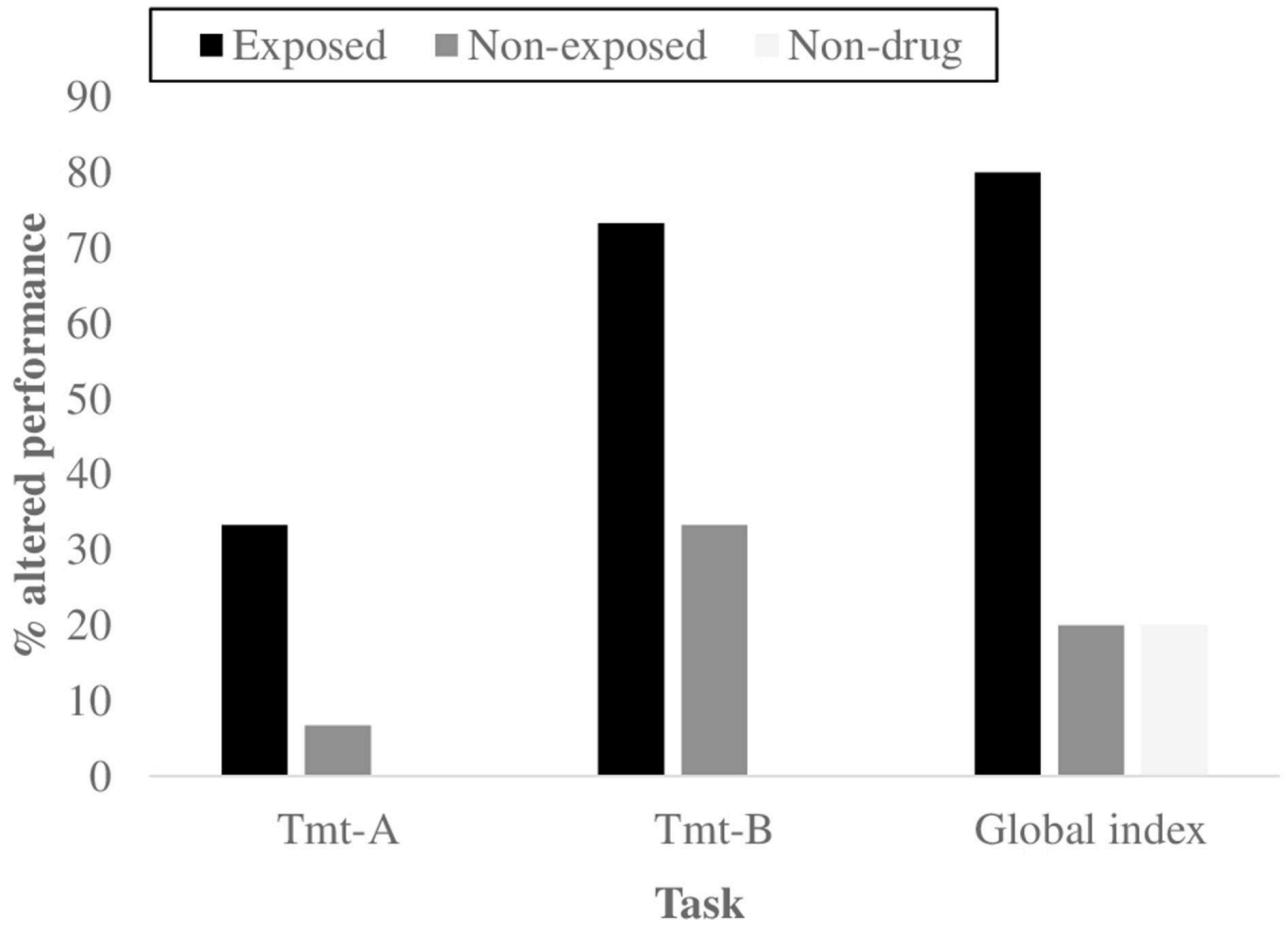
**Percentages of subjects with an altered global Cognitive profile and an altered performance for the two neuropsychological tasks that showed significant differences using Kruskal–Wallis and Mann–Whitney's U tests, as a function of group**.

Finally, to investigate the neuropsychological functioning and the potential presence of specific deficits among drug-addicted offspring of drug misusing parents, we first used the Kruskal–Wallis test to compare the z-scores: the 3 groups showed a different global cognitive profile (X^2^ = 13.570, *p* = 0.001). Moreover, a significant difference emerged for two measures of attention and executive function, the Trial Making Test-A (X^2^ = 13.178, *p* = 0.001) and -B (X^2^ = 14.674, *p* = 0.001), also according to Bonferroni correction (*p* = 0.05∕3=0.017) (Figure 2). The Mann–Whitney's U test reported that the exposed group showed a poorer performance in the Tmt-A and Tmt-B tests (*z* = −3.218, *p* = 0.001) (*z* = −3.780, *p* = 0.000) and a lower global cognitive profile (*z* = −2.826, *p* = 0.004) compared to the non-clinical group. The Pearson's Chi-square test confirmed the previous results concerning the presence/absence of deficits in the 3 abovementioned measures: Global cognitive index (X^2^ = 6.000, *p* = 0.042), Tmt-A (X^2^ = 17.368, *p* = 0.000) and Tmt-B (X^2^ = 10.800, *p* = 0.003). All these results remained significant according to the Bonferroni correction (*p* = 0.05∕3 = 0.017), with the only exception of Global cognitive index. With regard to the comparison between the non-clinical group and the non-exposed group, only the Tmt-B test showed significant results, for both the Mann–Whitney test (*z* = −3.332, *p* = 0.041) and the Pearson's Chi-square test (X^2^ = 6.000, *p* = 0.042).

### Comparing exposed and non-exposed groups

The two clinical groups were first compared in relation to the symptomatological profile at the SCL-90-R; neither the Mann–Whitney nor the Pearson's Chi-square tests reported significant differences between young drug addicts prenatally and/or postnatally exposed to parental SUDs and non-exposed drug-addicted inpatients. Similarly, a comparison of the personality assessment with the SWAP-200 procedure did not reveal specific characteristics in one of the two groups, with comparable levels of personality adaptive functioning and personality disorders and traits.

Instead, significant differences emerged in the neuropsychological functioning of the two groups of inpatients; individuals exposed to parental SUDs at the time of conception, intrauterine life and/or developmental age showed a poorer global cognitive profile for the Mann–Whitney test (*z* = −3.324, *p* = 0.002) and for the Chi-square test (X^2^ = 10.800, *p* = 0.003). Concerning the single tasks of the battery, the exposed group demonstrated more problems in the Tmt-A test (*z* = −3.032, *p* = 0.002). Results remained significant when the Bonferroni correction was used (*p* = 0.05∕2 = 0.025).

A *post hoc* power analysis was performed for the Chi square and Mann–Whitney tests; the *post hoc* power of the study (1−β) were 0.37 and 0.25 respectively, indicating that the effect is small and the sample size is small as well.

### Correlational analysis

Spearman's Rho was applied to test for any associations between the different measures adopted in the study. The main interest focused on potential associations between demographic measures that were relevant for the purposes of the research (the traumatic nature of being exposed to parental drug use) and psychological characteristics, in particular neuropsychological functioning. Results reported that the Global cognitive index was negatively correlated with having a parent with SUDs (*r* = −0.617^**^, *p* = 0.000), while the number of impaired EF was positively associated with it (*r* = 0.459^*^, *p* = 0.011). Importantly, the number of past traumatic experience (namely attachment disruptions and/or abuse) correlated positively with the number of executive impairments (*r* = 0.381^*^, *p* = 0.038). As expected, the level of education is inversely associated with executive performance (*r* = −0.392^*^, *p* = 0.032) and positively with the Global cognitive index (*r* = 0.440^*^, *p* = 0.015). No significant associations emerged between these two cognitive composite measures and personality or symptom assessments.

## Discussion

The present investigation reports preliminary results which indicate that young adults (aged 18–24) with SUDs showed a severe clinical condition; in particular, those who were exposed to parental alcohol and/or illicit drug use during their prenatal and/or postnatal development showed specific dysfunctional traits, compared with two groups of peers matched for age and level of education.

With regard to the preliminary analysis, while the symptomatological status could differentiate young people with SUDs from non-dependent young adults to some degree, the two clinical groups of inpatients had a high psychological distress at the moment of the assessment. On the basis of equal symptomatic distress, the examination of the personality profile with SWAP-200 indicated no significant characteristics in young drug addicts who were subject to drug-exposure compared to those who were not, suggesting that parents' drug issues may not have consequences for the development of specific personality traits or disorders. Research on personality of offspring of drug-dependent parents is mostly limited to personality characteristics and to children who have not developed a SUD themselves (Barr et al., 2006; Elkins et al., 2006; Morgan et al., 2010; Hinrichs et al., 2011), providing scarce data to compare with the present results. However, the lack of dissimilarity between the two groups suggests that personality disorders constitute the dysfunctional outcome of developmental trajectories that are not exclusively associated with parents' addiction problems. Rather, personality disorders (PDs) can be ascribed to a wide range of factors, including environmental variables which date back to childhood and adolescence, and which encompass maladaptive parenting, aversive interactive patterns with the primary caregivers, early adverse experiences and household dysfunction (Gibb et al., 2001; Afifi et al., 2011). The investigation of the role played by these factors goes beyond the aims of the present study, but further research should clarify this issue in the specific case of second-generation addicts. The high rates of personality disorders in our sample are in line with other (but still limited) studies specifically targeting young drug addicts, which estimate a prevalence of 46–61% (Kokkevi et al., 1998; Langås et al., 2012). On the one hand, our findings are consistent with research attesting a pervasiveness of Cluster B disorders among individuals with SUDs (Kokkevi et al., 1998; Mackesy-Amiti et al., 2012); this co-occurrence is explained by impulsivity, considered a common etiological process (Bornovalova et al., 2005; Krueger et al., 2007), On the other hand, it is worth mentioning that about 33% of the individuals in our group met the criteria for Dependent Personality as primary diagnosis, resembling other investigations which report Cluster C disorders in young substance users (Langås et al., 2012). The high percentage of Cluster C disorders, specifically Dependent PDs, might be explained by the application of a quite innovative assessment procedure, the SWAP-200. First, it relies on an external observer's judgment instead of self-reporting; secondly, it asks the clinician to base his/her judgments not only on the observation of manifest behaviors, but on some inference about internal psychological processes as well. Thus, the SWAP-200 assessment might have drawn attention to Dependent personality traits, otherwise overlooked.

Parental drug issues seemed to play a different role concerning the neuropsychological functioning of young-adult offspring with SUDs; those exposed to childrearing environments affected by addiction (prenatally and/or postnatally) showed more cognitive impairments, especially at the expense of EF, in terms of attention shifting and mental flexibility. From a qualitative point of view, the executive domains of cognitive estimation, planning and memory also displayed an inadequate performance. Interestingly, the data are similar to those reported by Warner et al. (2006). Executive function deficits are particularly critical, given their role for individual adjustment and emotional competences (McClelland et al., 2007; Blair and Ursache, 2011). The presence of reduced higher-order cognitive efficiency in the group of drug-addicted offspring can be explained referring to the contribution of multiple interacting factors. First, a vast literature attests that prenatal drug exposure leads to subtle but enduring consequences for the prefrontal cortex and can cause cognitive impairments in childhood, adolescence (e.g., Warner et al., 2006; Rose-Jacobs et al., 2011) and young adulthood, even if research is more restricted in this developmental stage (Kerns et al., 1997; Fried and Smith, 2001; Smith et al., 2006). Considering these established long-lasting and harmful effects, drug taking during pregnancy can be considered a form of maltreatment and trauma, which occur extremely early and function as an early matrix for later maladaptive developmental trajectories. Second, being raised in an adverse and deprived environment (with parents scarcely involved in promoting their child's education and providing environmental stimulation) and having experienced early traumatic events (often of an interpersonal nature) are factors capable of compromising optimal cognitive development (Mayes, 2002; El-Sheikh and Buckhalt, 2003; Bennett et al., 2008; Singer et al., 2008). Eventually, if previous impairments of the executive and control abilities can predispose to substance use (Giancola and Tarter, 1999), it is also proven that drug misuse during the highly sensitive adolescent years (Chambers and Potenza, 2003) has a detrimental effect on cognitive abilities (Tapert et al., 2002; Hanson et al., 2010; Solowij and Pesa, 2010).

Although the present study has some strengths (addressing a very specific clinical group and not relying on self-reporting for personality assessment), the current results should be interpreted relative to some limitations, which underlie the preliminary nature of the research.

The major limitation regards the small sample size; as a matter of fact, power analyses revealed that the effect is small and the sample size is small as well; future studies will necessitate a sample size that exceeds the currently available resources.

Second, the cross-sectional nature of the study does not allow conclusions on the causality link between the examined factors; in order to clarify this association, other research designs could provide more accurate information; for example, a longitudinal approach would be extremely useful to better understand the developmental pathways in offspring at risk because of parental mental health problems. However, this type of studies shows several challenges in feasibility and sometimes might be not possible at all. Alternatively, innovative statistical and mathematical methods may constitute a valuable alternative approach for future research, as suggested by Maurage et al. (2013).

Other limitations include the use of dichotomies for the consideration of parental substance misuse and the lack of distinction among different drugs used by parents and young addicts (alcohol, cocaine, heroin, marijuana); moreover, further research should adopt an accurate control of important variables such as gender, socio-economic status, type of traumatic experiences. It has been recognized that other relevant factors are capable of compromising optimal cognitive functioning (Mezzacappa, 2004; Bennett et al., 2008; Dikmen et al., 2009; Evans and Schamberg, 2009; Hackman and Farah, 2009). Thus, future studies might investigate the role of childrearing environment characteristics on the neuropsychological functioning, since the present research did not examine these aspects.

In conclusion, the preliminary results of this study have pointed out that parental drug use could be an additional factor that makes the clinical condition of young drug addicts particularly severe (especially in terms of neuropsychological impairments), contributing to the currently limited knowledge on young-adult children of drug-dependent parents, with SUDs themselves. Replication of the present data is necessary in order to increase confidence in results and to better guide clinical implications that the study has preliminarily indicated. In particular, a strong need would emerge for multi-focused prevention and intervention programs for young adults with SUDs in general, and specifically for second-generation addicts. First, assessment and treatment programs are required to target drug-addicted mothers and their at-risk children as early as the prenatal and early postnatal periods (Stocco et al., 2012; De Palo et al., 2014). With regard to EF, early prevention and educational protocols should sustain cognitive development of children of drug-addicted parents (Minnes et al., 2014), in order to buffer their vulnerability and prevent future detrimental outcomes. Recent studies indicate that specific trainings, such as mindfulness, can ameliorate executive function even in adulthood (Posner et al., 2014). Concerning young adults with established SUDs, neurocognitive deficits are likely to interfere with treatment and recovery (Aharonovich et al., 2006; Grant et al., 2013); as a consequence, the introduction of protocols targeting cognitive functions is highly recommended. Moreover, programs for drug-addicted offspring of parents with SUDs should be augmented with an integrative approach that combines trauma-focused work and that adopts a developmental perspective, bridging the gap between childhood and outcomes in early adulthood. Despite a growing awareness of the need for trauma-informed substance abuse treatment (Brown et al., 2008; Taplin et al., 2014), nowadays there are few intervention programs addressing complex trauma experiences that pre-exist/co-exist with SUDs in young adulthood (Rosenkranz et al., 2014), requiring further development.

## Author contributions

MP, AS, DM, MS and PC have given a substantial contribution to the ideation and implementation of the work, taking part to data acquisition, analysis, and interpretation, drafting and revising the manuscript, and reaching an agreement on the final version of the work.

### Conflict of interest statement

The authors declare that the research was conducted in the absence of any commercial or financial relationships that could be construed as a potential conflict of interest.

## Footnotes

^1^Subjects of the study were part of a larger research protocol approved by the Ethic Commette of the University of Padova
